# Evaluation of the anatomical locations and sizes of uterine fibroids from ultrasound examination in Ghana

**DOI:** 10.4314/ahs.v23i4.48

**Published:** 2023-12

**Authors:** Emmanuel Kobina Mesi Edzie, Klenam Dzefi-Tettey, Edmund Kwakye Brakohiapa, Philip Narteh Gorleku, Eric Aidoo, Stephen Kwaku Amoah, Samuel Asiamah, Frank Quarshie, Obed Nimo, Amrit Raj, Joshua Mensah Kpobi, Evans Boadi, Richard Ato Edzie, Veronica Turkson, Henry Kusodzi, Abdul Raman Asemah

**Affiliations:** 1 Department of Medical Imaging, School of Medical Sciences, College of Health and Allied Sciences, University of Cape Coast, Cape Coast, Ghana; 2 Department of Radiology, Korle Bu Teaching Hospital, 1 Guggisberg Avenue, Accra, Ghana; 3 Department of Radiology, University of Ghana Medical School, Accra, Ghana; 4 Department of Anatomy, School of Medical Sciences, College of Health and Allied Sciences, University of Cape Coast, Cape Coast, Ghana; 5 African Institute for Mathematical Sciences (AIMS), Summerhill Estates, East Legon Hills, Santoe, Accra, Ghana; 6 Department of Imaging Technology and Sonography, College of Health and Allied Sciences, University of Cape Coast, Cape Coast, Ghana; 7 Department of Paediatrics, School of Medical Sciences, College of Health and Allied Sciences, University of Cape Coast, Cape Coast, Ghana; 8 Department of Radiology, Cape Coast Teaching Hospital, Cape Coast, Ghana

**Keywords:** Uterine fibroids, Anatomical locations, Sizes, Ultrasound imaging, Ghana, Africa

## Abstract

**Background:**

Uterine fibroids locations and sizes, have significant influences on the quality of life of women especially pregnant women and on live birth rate.

**Objectives:**

To determine the anatomical locations and sizes of uterine fibroids and assess any possible associations with age groups.

**Methods:**

This retrospective study reviewed the locations and sizes of a total of 3,542 patients who were ultrasonographically diagnosed with uterine fibroids from January 2019 to December 2021. The obtained data were analysed using GNU PSPP, and Python on Jupyter Notebook with statistical significance level set at p≤0.05.

**Results:**

The overall average diameter of uterine myoma was 5.50±2.60cm (95%CI=5.41–5.58, range=1.00cm–19.10cm) and the respective mean diameter of intramural, subserosal and submucosal fibroids were 5.53±2.60cm (95%CI=5.44–5.62), 5.50±2.27cm (95%CI=5.27–5.74) and 5.82±2.77cm (95%CI=5.49–6.14). Most of the fibroid nodules were small (48.36%, n=1713) and only 5.84% (n=207) were large (>10cm). Posterofundal fibroids and lower anterior myomas were mostly seen in the 35-44 years age class.

**Conclusion:**

The majority of the uterine fibroids were intramural and were mostly at the anterior uterine wall. The submucosal fibroids, even though the rarest, were averagely larger than the other types of uterine myomas. The diameter of uterine fibroids increased with age.

## Introduction

Uterine myomata are the most common smooth muscle benign tumors of the uterus, comprising varying levels of fibrous tissues and smooth muscle cells.^1^ They compress the surrounding connective tissues and the myometrium, causing the progressive development of collagen rich pseudocapsules, blood vessels, and neurofibres. A recent study reported that, uterine fibroids have adverse effects on the reproductive system, due to significant gynaecological morbidity. In Australia and the United States of America (USA) for instance, literature has reported that myomata are the most common indication for hysterectomy, in 40-60% of all cases.^2^

Studies have shown that, during the reproductive years of women, the majority of them will develop uterine fibroids. In the USA, it is estimated that, about 26 million women between the ages of 15 and 50 years have uterine myomas, with records of high impacts, prevalence and incidence in the black community. African American women, on average, are younger at first diagnoses of fibroids, with larger and more multiple tumours, and a higher likelihood of myomectomy.^3^ The total annual fibroids hospital admission cost in England, Germany and France were $86 million, $120 million, and $348 million respectively, and these especially impact the black community.^1^

Most patients with fibroids are asymptomatic as only 30% presents with symptoms like infertility, constipation, urinary frequency, anaemia, and abnormal uterine bleeding. Routine gynaecological screening, bleeding per vaginum, menorrhagia, amenorrhoea, dysmenorrhoea, and pregnancy are common indications for pelvic ultrasonography that aid in the diagnosis of uterine fibroids.^1^,^4^

When uterine fibroids are suspected, ultrasound is the standard imaging examination owing to its high specificity and sensitivity in diagnosing this condition. The ultrasound scans can be performed transabdominally (transabdominal scan – TAS) or transvaginally (transvaginal scan – TVS). Despite both TAS and TVS can be used in most situations of pelvic diseases, the transvaginal sonography is preferable to transabdominal sonography.^5^ Magnetic resonance imaging (MRI) is the gold standard for the diagnosis of uterine fibroids as it can more accurately localise and quantify the fibroid nodules. However, ultrasonography is usually the main modality for diagnosis of uterine fibroids in most developing countries like Ghana because of its availability and affordability.^6^,^7^

The International Federation of Gynaecology and Obstetrics (FIGO) proposed the allocation of fibroids into seven types, from type 0 (where the submucosal fibroid is completely enclosed within the uterine cavity or pedunculated intracavitary fibroid) to type 7 (where inside the pelvis lies a pedunculated fibroid), aiming to arrive at a universal and detailed classification, but to date, there is no universally accepted way of classifying uterine fibroids. Uterine myomas are mostly categorised according to their anatomical relationship to the endometrium, and myometrium. Thus, currently, the location is the only and basic feature for their classification resulting in three topographic categories: subserosal, intramural, and submucosal fibroids.^8^,^9^

The locations and sizes of uterine leiomyomata help to determine their clinical behaviour, yet such studies are limited in our setting. Other studies have reported on the anatomical locations and sizes of fibroids with intramural as the most common location for fibroid, but only few assessed possible associations between the locations, sizes and age groups.^4^,^10^,^11^ We sought to determine the anatomical locations and sizes of fibroids, and assess any possible associations with age groups. The specific objectives were;

* To assess the anatomical locations of uterine fibroids.
* To ascertain the associations between the anatomical locations of uterine myoma and the age groups.
* To assess the sizes of uterine fibroids and possible associations with age groups.

## Methods

### Study setting and design

The ultrasound scan records and ages of all participants who visited the Cape Coast Teaching Hospital (CCTH) from January 2019 to December 2021 were evaluated in this retrospective cohort study. The CCTH is the only public tertiary facility serving the South-Central Ghana populace, and it is situated in Cape Coast, the regional capital. The facility is one of Ghana's top medical research institutions and a training site for the College of Health and Allied Sciences of the University of Cape Coast and the Nursing-Midwifery Training College of Cape Coast. It provides tertiary and subspecialty services (including radiology) to the region and beyond. The ages of the participants were categorised as follows; “15-24 years”, “25-34 years”, “35-44 years”, “45-54 years”, and “55-69 years”. The sizes (in diameter) of the fibroid nodules were also classified as follows; “small (<5 cm)”, “medium (5-10 cm)”, and “Large (>10 cm)”.^12^ The locations were themed under, the types; intramural, submucosal and subserosal; and the positions; “anterior uterine fundus”, “anterior uterine body”, “anterior lower uterus/cervix”, “posterior uterine fundus”, “posterior uterine body”, “posterior lower uterus/cervix” and “central uterine fundus”.

### Data collection

Over the three-year period, we assessed the number of patients diagnosed with uterine fibroids by ultrasonography since ultrasonography is commonly used to diagnose uterine myomas because of its radiation-free nature, affordability, availability, high specificity, and sensitivity.^13^ We employed the Lightwave Health Information Management System (LHIMS), a computerised database system of the Cape Coast Teaching Hospital (CCTH), which has been used for many years and contains a detailed patient's information on height, weight, demographics, diagnosis, laboratory results, procedures as well as the radiology reports. Keywords like uterine myomas, fibroids, leiomyomas, hysterectomy, and myomectomy were used to find all the patients who had been diagnosed with uterine fibroids. After that, their unique electronic identification numbers were obtained to avoid duplication. All linked automated data sources of the patients were retrieved using the unique electronic identification number generated. We conducted a detailed review of the medical records to extract the ultrasound scan fibroid reports for the anatomical locations and sizes based on the categorised themes, as well as the age groups of the patients. For analyses, a total of 3,542 patients diagnosed with uterine fibroids were consecutively selected and included.

### Image acquisition

The ultrasound scans were performed by three radiologists with over 10 years of cumulative experience in gynaecological sonography, using Mindray Diagnostic Digital Ultrasound System, Model DCN3, with a 3.5MHz convex probe and a 7.5MHz transvaginal probe, manufactured in 2014 by Shenzhen Mindray Bio-Medical Electronics Company Limited (Nanshan, Shenzhen, China). The examinations were done in transverse and sagittal planes to locate the uterine fibroids and measure their sizes (in centimeters).

### Statistical analysis

GNU PSPP (Category: Education, Science & Math), pspp version 1.2.0-3, developed by the Free Software Foundation, was used for collating, grouping, inputting, and analysing the data to obtain the tables, frequencies and percentages. The charts were created using Python on Jupyter Notebook (Version 3.0) and LibreOffice Calc (version 1:6.1.5-3+deb10u6), developed by The Document Foundation. A Chi-square test was used to examine for possible associations between age and anatomical locations and sizes of the uterine fibroids. P-values ≤ 0.05 were chosen to be statistically significant.

## Results

The overall mean size (in diameter) of the fibroid masses was 5.50±2.60cm ranging from 1.00cm to 19.10cm and the average age of the patients was 35.58±9.04 years, also spanning from 17-68 years. The age group with the highest prevalence was the 25-34 years (40.49%) and the least was 55-69 years (3.53%). The majority of the patients had small fibroid sizes (48.36%, n=1713), followed by medium sizes (45.79%, n=1622). Only 5.84% (n=207) had large fibroid sizes as shown in (Table 1).

**Table 1:** Age distribution and sizes of the fibroid nodules of the patients

Variable	Count	Percentage (%)
**Age;**		
Minimum	17 years	
Maximum	68 years	
Mean (SD)	35.58 (9.04) years	



**Age Group;**		
15-24 years	318	8.98%
25-34 years	1434	40.49%
35-44 years	1193	33.68%
45-54 years	472	13.33%
55-69 years	125	3.53%

**Fibroid Size;**		
Minimum	1.00cm	
Maximum	19.10cm	
Mean (SD)	5.50 (2.60) cm	



**Fibroid Size Categories;**		
Small (<5cm)	1713	48.36%
Medium (5-10cm)	1622	45.79%
Large (>10cm)	207	5.84%

Abbreviation: SD, Standard Deviation.

The mean ages of the patients who had small, medium and large fibroid sizes were 32.52 years (CI=32.12–32.91), 36.99 years (CI=36.61–37.37) and 49.84 years (CI=48.90–50.78) respectively. The mean ages for those who had intramural, subserosal and submucosal fibroids were 35.30 years (CI=35.00– 35.60), 35.55 years (CI=34.62–36.47) and 38.55 years (CI=37.48–39.63) respectively. The average and diameters/sizes of the intramural, subserosal and submucosal uterine fibroids are shown in (Table 2).

**Table 2:** Mean ages, fibroid sizes and fibroid location catagories of patients

Variable	Mean Age (SD)	95% CI for Mean	F-value	P-value
Small (<5cm)	32.52 (8.37) years	32.12 – 32.91	476.93	<0.001*
Medium (5-10cm)	36.99 (7.77) years	36.61 – 37.37		
Large (>10cm)	49.84 (7.12) years	48.90 – 50.78		
Overall	35.58 (9.04) years	35.28 – 35.88		

Intramural	35.30 (8.68) years	35.00 – 35.60	6.01	0.014*
Subserosal	35.55 (8.93) years	34.62 – 36.47	0.08	0.771
Submucosal	38.55 (9.16) years	37.48 – 39.63	38.79	<0.001*
	**Mean Size (SD)**			
Small (<5cm)	3.42 (0.98) cm	3.37 – 3.47	6640.69	<0.001*
Medium (5-10cm)	6.87 (1.33) cm	6.81 – 6.94		
Large (>10cm)	11.87 (1.58) cm	11.66 – 12.09		
Overall	5.50 (2.60) cm	5.41 – 5.58		

Intramural	5.53 (2.60) cm	5.44 – 5.62	7.50	0.006*
Subserosal	5.50 (2.27) cm	5.27 – 5.74	0.00	0.965
Submucosal	5.82 (2.77) cm	5.49 – 6.14	4.67	0.031*

Abbreviation: SD, Standard Deviation; CI, Confidence Interval

* Statistically Significant

**Figure 1:**
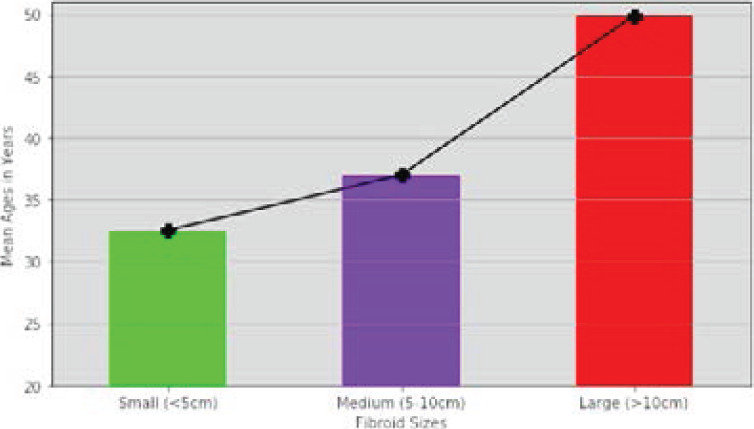
Fibroid sizes and the respective mean ages of the patients

Figure 1 depicts a direct relationship between fibroid sizes and mean ages. Hence, the sizes of fibroid nodules, on the average, increased with age.

**Figure 2:**
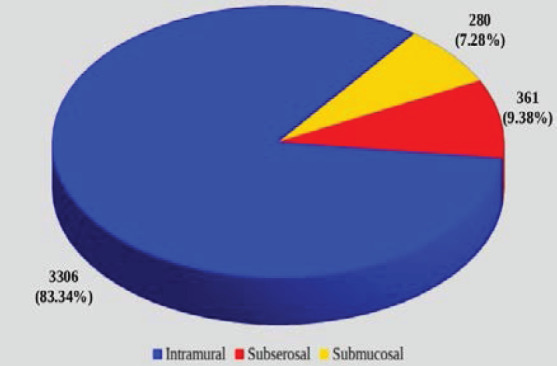
The proportions of the various types of uterine fibroids

The most frequent type of uterine fibroids was the intramural (83.34%, n=3306) followed by subserosal (9.38%, n=361) and submucosal (7.28%, n=280) (Figure 2).

**Figure 3:**
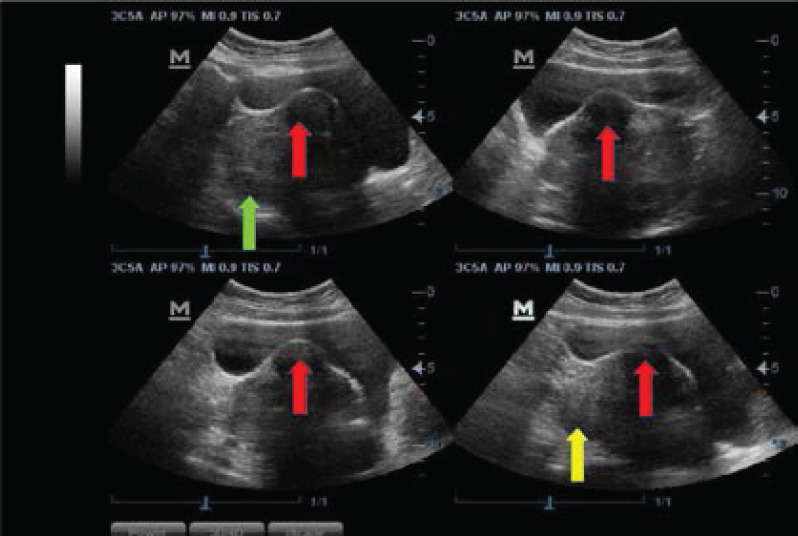
Sonograms showing anterior intramural uterine body myoma indenting into the posterior wall of the urinary bladder (red arrows), posterior uterine body myoma (green arrow), and a posterior uterine fundus (yellow arrow)

**Figure 4:**
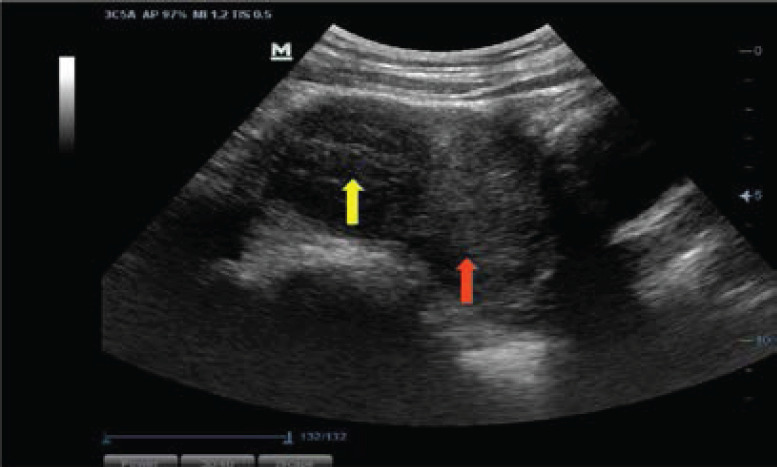
A longitudinal ultrasound image of the uterus showing a centrally fundal myoma (yellow arrow) and a posterior lower uterine myoma (red arrow)

**Figure 5:**
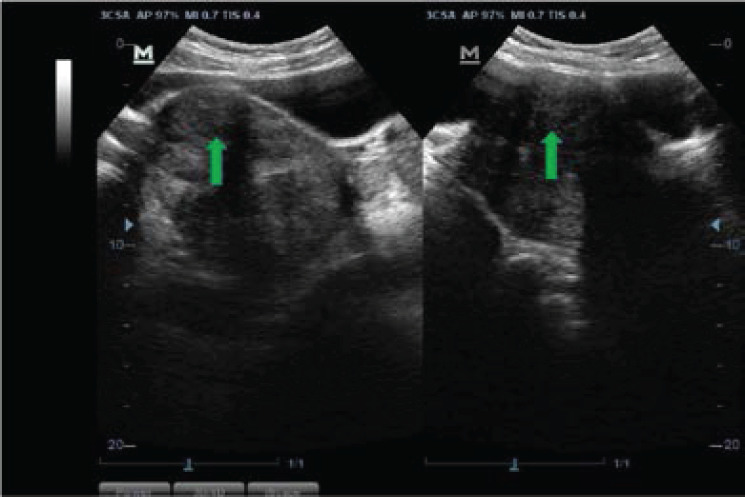
Ultrasound images of the uterus showing a myoma at the anterior uterine fundus (green arrows)

**Figure 6:**
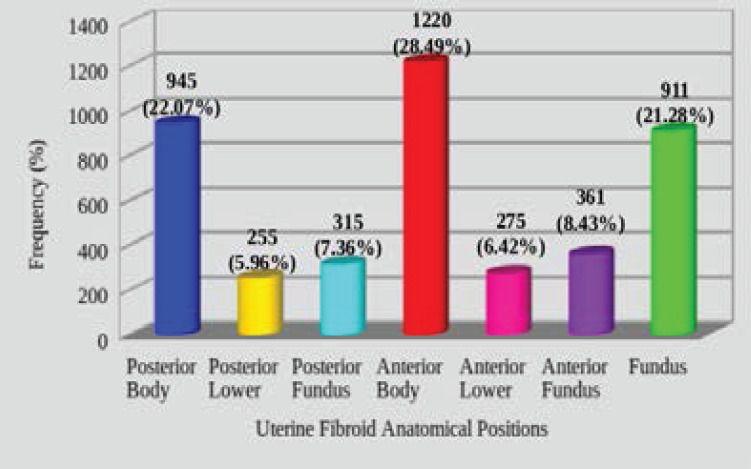
The proportions of the anatomical positions of uterine fibroids

Most of the fibroid masses were anatomically positioned at the anterior part of the uterine body (28.49%, n=1220) (Figure 3), and posterior part of the uterine body (22.07%, n=945) (Figure 3), followed by the centrally fundal part (21.28%, n=911) (Figure 4) and anterior uterine fundus (8.43%, n=361) (Figure 5). Only 5.98% (n=255) of the fibroid masses were at the posterior lower part of the uterus (Figure 4). The rest are shown in (Figure 6).

Most of the intramural fibroids were located at the anterior part of the uterine body (29.29%, n=1150) and only 5.86% (n=230) were at the posterior lower part of the uterus. The subserosal and submucosal fibroids were both more common at the fundal part of the uterus constituting 32.53% (n=176) and 29.11% (n=115) respectively. Most of the small fibroids were at the anterior regions (45.31%) with the following specific constituents: body 35.75%; lower 0.54%; and fundus 9.02%. The remaining distributions of the types and sizes of uterine fibroids at the various anatomical locations or positions are showed in (Table 3).

**Table 3:** Distribution of the uterine fibroids types and sizes at the various locations

Location	Intramural	Subserosal	Submucosal	<5cm	5-10cm	>10cm
Posterior Uterine body	885 (22.54%)	95 (17.56%)	60 (15.19%)	466 (25.01%)	402 (19.55%)	77 (28.84%)
Posterior lower uterus	230 (5.86%)	25 (4.62%)	50 (12.66%)	89 (4.78%)	135 (6.57%)	31 (11.61%)
Posterior uterine fundus	305 (7.88%)	20 (3.70%)	20 (5.06%)	122 (6.55%)	168 (8.17%)	25 (9.36%)
Total posterior	1420 (36.28%)	140 (25.88%)	130 (32.91%)	677 (36.34%)	706 (34.29%)	133 (49.81%)
Anterior uterine body	1150 (29.29%)	165 (30.50%)	55 (13.92%)	666 (35.75%)	528 (25.68%)	26 (9.74%)
Anterior lower uterus	250 (6.37%)	30 (5.54%)	60 (15.19%)	10 (0.54%)	153 (7.44%)	16 (5.99%)
Anterior uterine fundus	326 (8.30%)	30 (5.54%)	35 (8.86%)	168 (9.02%)	166 (8.07%)	27 (10.11%)
Total anterior	1726 (43.96%)	225 (41.48%)	150 (37.97%)	844 (45.31%)	847 (41.19%)	69 (25.84%)
Central uterine fundus	780 (19.87%)	176 (32.53%)	115 (29.11%)	342 (18.36%)	504 (24.51%)	65 (24.34%)

**Figure 7:**
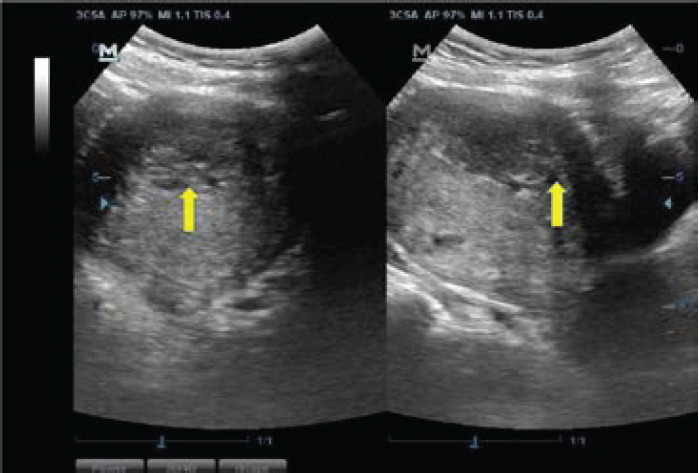
An ultrasound scan of the uterus showing a submucosal predominantly hypoechoic anterior myoma

The majority of the patients with intramural fibroids were in the 25-34 years (40.36%, n=1294) and 35-44 years (35.09%, n=1125) age categories (p=0.001). Similar distribution across the age groups was seen in the patients with subserosal fibroids with p-value=0.003. The submucosal fibroids (Figure 7) were more frequent in the 35-44 years (39.29%, n=110) and 25-34 years (32.14%, n=90) age groups (p<0.001). The remaining distribution of the anatomical positions of the uterine fibroids across the various age classes are shown in (Table 4). The fibroids with small sizes were more common (62.87%) in the relatively younger population (≤34 years) whilst the large ones were more frequent (60.68%) in the older patients (≥45 years). The rest are shown in (Table 4).

**Table 4:** Association between the anatomical locations, sizes and age groups of uterine fibroids

Fibroid Type	15-24years	25-34years	35-44years	45-54years	55-69years	P-value
Intramural	293 (9.14%)	1294 (40.36%)	1125 (35.09%)	420 (13.10%)	74 (2.31%)	0.001*
Subserosal	15 (4.16%)	165 (45.71%)	125 (34.63%)	42 (11.63%)	14 (3.88%)	0.003*
Submucosal	10 (3.57%)	90 (32.14%)	110 (39.29%)	55 (19.64%)	15 (5.36%)	<0.001*
**Position;**						
Posterior uterine body	65 (6.88%)	356 (37.67%)	352 (37.25%)	144 (15.24%)	28 (2.96%)	0.004*
Posterior lower uterus	25 (9.80%)	100 (39.22%)	70 (27.45%)	55 (21.57%)	5 (1.96%)	0.001*
Posterior uterine fundus	45 (14.29%)	110 (34.92%)	115 (36.51%)	40 (12.70%)	5 (1.59%)	0.004*
Total posterior	135 (9.09%)	566 (38.12%)	537 (36.16%)	209 (14.07%)	38 (2.56%)	
Anterior uterine body	83 (6.80%)	567 (46.48%)	445 (36.48%)	95 (7.79%)	30 (2.46%)	<0.001*
Anterior lower uterus	25 (9.09%)	85 (30.91%)	110 (40.00%)	45 (16.36%)	10 (3.64%)	0.014*
Anterior uterine fundus	75 (20.78%)	160 (44.32%)	90 (24.93%)	31 (8.59%)	5 (1.39%)	<0.001*
Total anterior	183 (9.86%)	812 (43.75%)	645 (34.75%)	171 (9.21%)	45 (2.42%)	
Central uterine fundus	40 (4.39%)	351 (38.53%)	313 (34.36%)	172 (18.88%)	35 (3.84%)	<0.001*
**Size;**						
Small (<5cm)	291 (16.99%)	786 (45.88%)	487 (28.43%)	125 (7.30%)	24 (1.40%)	<0.001*
Medium (5-10cm)	25 (1.54%)	644 (39.70%)	672 (41.43%)	235 (14.49%)	46 (2.84%)	
Large (>10cm)	2 (0.97%)	4 (1.93%)	34 (16.43%)	112 (54.11%)	55 (26.57%)	

* Statistically Significant

## Discussion

The locations and sizes of uterine fibroids according to literature, have significant influences on the quality of life of women especially pregnant women and on live birth rate (proportion of babies that are born alive). Studies have reported that fertility may be impaired by fibroids (submucosal and intramural myomas extending towards the endometrial lining).^14^,^15^ Even though intramural fibroids, the commonest type of uterine fibroids (83.34%) in this current study, are generally not dangerous, they can cause unpleasant symptoms and may contribute to subfertility (Figure 2) as also corroborated by several other studies.^8^,^16^ The subserosal (9.38%) and submucosal (7.28%) fibroids were the second and the least common types of fibroids respectively in this study. A similar picture of finding of intramural, subserosal, and submucosal in their respective order of frequencies has been reported by other studies.^17^,^18^ A study by Moshesh et al., in Michigan, USA recorded a margin of 79.0% for the intramural fibroids and a comparatively low proportion of 4.0% for the submucosal fibroids.^17^ In a rural population of North India, Dayal et al., also reported intramual myomas as the commonest (66.7%), followed by subserosal (22.2%) and submucosal (8.9%).^18^ Padubidri et al., also found intramural fibroids to be the most predominant (75.0%) but contrary to what we found, submucosal (15.0%) was the second most frequent type of uterine leiomyomata, followed by subserosal (10.0%) in their study.^19^

The themes of uterine fibroids locations vary across studies. In this current study, the anterior and posterior locations of uterine fibroids were further categorised as body, lower and fundal but generally, the majority of the uterine fibroids were anatomically positioned in the anterior wall (43.34%), and posterior uterine wall (35.39%) (Figure 6). Zhang et al., broadly categorised the positions as posterior (44.2%), anterior (34.0%) and others (21.8%) thereby recording most of the fibroids at the posterior uterine wall, which is contrary to our findings.^14^ This may give some advantages in terms of treatment to women with fibroids in our setting as literature has reported that fibroids located at the anterior wall of the uterus are less difficult to treat especially with ultrasound-guided high intensity focused ultrasound (HIFU) ablation than those located at the posterior wall.^20^ Another study by Adesina et al., also reported that, fibroids at the anterior wall mostly require anterior uterine incisions which are associated with least amount of blood loss as compared to those at the posterior wall mostly requiring posterior uterine incisions, which are significantly associated with increased blood loss.^21^

Most of the uterine fibroid nodules in this study were of relatively smaller sizes thus less than 5cm (48.36%), followed by medium sizes (5-10cm, 45.79%) and large sizes (>10cm, 5.84%) (Table 1). Similar results have been reported by Sarkodie et al., but with a comparatively higher proportion of 64.4% of the fibroid masses less than 5cm.^22^ In another study by Lee et al., divergent results were observed. The authors recorded the majority of the uterine myoma nodules being medium (5-10cm, 48.11%), followed by small (<5cm, 42.45%) and large (>10cm, 9.44%).^12^ The mean diameter of uterine fibroids differ across studies and jurisdictions even though generally literature posits that leiomyomas are larger in Africans or African American women.^3^,^23^

The overall mean diameter of uterine fibroids in our study was 5.50±2.60cm (range: 1.00-19.10cm), which may pose some threats on the quality of life of women in our setting particularly pregnant women (Table 1). According to literature, pregnant women with fibroid size s>3cm have high risk of developing complications even though they may deliver at a significantly earlier gestational age as compared to women without fibroids.^24^,^25^ Similar average diameter of 5.4cm (range: 1.6-18.2 cm) was reported by Deipolyi Amy.^26^ A comparatively higher mean diameter of 8.3cm was found by Katsumori et al., in Japan and 4.1±0.8cm (range: 0.7-10.7cm) by Ciavattini et al., in their study which assessed the numbers and sizes of uterine fibroids and obstetric outcomes in Italy.^24^,^27^ Another study by Oliveira et al., in Brazil recorded an overall mean diameter of 1.9±1.3cm (range: 0.4- 6.9cm), but the reason for the significantly low average diameter could be due to the total exclusion of fibroids > 7cm from their study.^28^

The corresponding mean diameters of the intramural, subserosal and submucosal uterine fibroids in this current study were 5.53cm, 5.50cm and 5.82cm respectively, purporting that comparatively, the submucosal fibroids were larger, followed by intramural fibroids (Table 2). Similar results have been documented by Verma et al., who also recorded larger submucosal fibroids with a comparatively higher mean diameter of 8.2cm, but contrary to ours, their subserosal fibroids were the next largest.^29^ This could be due to the tendency of submusosal fibroids to grow bigger and even invade further into the uterine layers. A study by Bettocchi et al., reported that submucosal myomas can grow larger and infiltrate deeper into the uterine tissues, thereby posing a greater threat to fertility and making removal more difficult.^30^ Hence, from this present study, the relatively smaller submucosal fibroids is a good finding with regards to fertility outcomes and ease of surgical intervention.

The two top anatomical positions of intramural fibroids were at the anterior part of the uterine body (29.29%), and posterior part of the uterine body (22.54%), whilst the subserosal fibroids were most commonly positioned at the fundal region of the uterus (32.53%) and anterior part of the body (30.50%). In the study by Oliveira et al., the authors recorded the majority of intramural and subserosal fibroids at the fundus with comparatively higher proportions of 68.0% and 66.0% respectively.^28^ The occurrence of the anatomical locations of fibroid nodules in our setting may not have dramatic implications on fertility outcomes and make surgical intervention like HIFU ablation easier.

Unlike the intramural and subserosal fibroids which were more common in the comparatively younger age category (25-34 years) constituting 40.36% and 45.71% respectively, the submucosal fibroids recorded high prevalence in the late reproductive age group (35-44 years, 39.29%). This also reflected in the comparatively higher mean age for the submucosal fibroids as against the subserosal and intramural fibroids. The average age for submucosal fibroids was 38.55±9.16 years in this paper. Verma et al., reported a higher submucosal fibroid mean age of 42 years.^29^

We found that posterofundal fibroids (Figure 3) and myomas at the lower part of the anterior wall were more frequent in the 35-44 years age class, whilst the rest were predominantly in the 25-34 years group, both within the active reproductive age of women (15-49 years), which is in agreement with the pertinent literature.^31^ Small fibroids (<5cm) were comparatively more common in the younger age group (<35 years), also corroborated by Sarkodie et al.^22^

Generally, the sizes of fibroids (in diameter) increased with age (Figure 1), as reported in literature.^31^ The majority of the fibroids ≥5cm were at the anterior wall of the uterus, also corroborated by Shavell et al., in Detroit, USA. The authors further reported that such myomas (≥5cm) are commonly and significantly associated with preterm delivery, premature rupture of membranes, and short cervix coupled with high blood loss with requisite postpartum blood transfusion and attention.^32^ Even though numerous treatment options for fibroids are available based on the symptomatologies, locations, numbers, and sizes, they are limited for pregnant women, as some treatment alternatives may not be appropriate. For instance, prophylactic myomectomy is not recommended for pregnant women with fibroid size >5cm owing to its conferment of worse outcomes at delivery.^33^ Also, biochemical evidence generally suggests that the differences in oestrogen receptor densities in the different types of fibroids and the endometrium determines the frequency and distribution of the various types. Oestrogens upregulate oestrogen dependent growth of uterine fibroids while progesterone down regulates these actions after the cell mutations underlying their tendencies to over proliferate, have been established. The future holds promise for using hormonal manipulations for controlling the growth of uterine fibroids especially as the incidence rate is increasing in our setting as reported in a current study.^34^,^35^

## Limitation

The sample size for this study could have been under-estimated because patients whose information were not found on the LHIMS were not included.

## Conclusion

The majority of the uterine fibroids were intramural and mostly at the anterior uterine wall. Even though the submucosal fibroids were less common, they were averagely larger than the other types of uterine myomas and mostly seen at the central uterine fundus. The diameters of uterine fibroids increased with age. Fibroid nodules less than 5cm were mostly at the anterior uterine body whilst those greater than 10cm were common at the posterior uterine body.

## Data Availability

Access to the data used for this paper could be made available upon a formal request to the director of research, CCTH through the email address: ccthresearch@ gmail.com.
